# Machine Learning Predicts Pathologic Complete Response to Neoadjuvant Chemotherapy for ER+HER2- Breast Cancer: Integrating Tumoral and Peritumoral MRI Radiomic Features

**DOI:** 10.3390/diagnostics13193031

**Published:** 2023-09-23

**Authors:** Jiwoo Park, Min Jung Kim, Jong-Hyun Yoon, Kyunghwa Han, Eun-Kyung Kim, Joo Hyuk Sohn, Young Han Lee, Yangmo Yoo

**Affiliations:** 1 Department of Radiology and Research Institute of Radiological Science, Severance Hospital, Yonsei University College of Medicine, Seoul 03722, Republic of Korea; classic0610@yuhs.ac (J.P.); yoongh@yuhs.ac (J.-H.Y.); khhan@yuhs.ac (K.H.); oncosohn@yuhs.ac (J.H.S.); sando@yuhs.ac (Y.H.L.); 2 Department of Radiology, Research Institute of Radiological Science, Yongin Severance Hospital, Yonsei University College of Medicine, Yongin-si 06230, Republic of Korea; ekkim@yuhs.ac; 3 Department of Electronic Engineering, Sogang University, Seoul 04107, Republic of Korea; ymyoo@sogang.ac.kr

**Keywords:** ER+HER2- locally advanced breast cancer, neoadjuvant chemotherapy, pathological complete response, pretreatment MRI, segmentation, machine learning, radiomics

## Abstract

Background: This study aimed to predict pathologic complete response (pCR) in neoadjuvant chemotherapy for ER+HER2- locally advanced breast cancer (LABC), a subtype with limited treatment response. Methods: We included 265 ER+HER2- LABC patients (2010–2020) with pre-treatment MRI, neoadjuvant chemotherapy, and confirmed pathology. Using data from January 2016, we divided them into training and validation cohorts. Volumes of interest (VOI) for the tumoral and peritumoral regions were segmented on preoperative MRI from three sequences: T1-weighted early and delayed contrast-enhanced sequences and T2-weighted fat-suppressed sequence (T2FS). We constructed seven machine learning models using tumoral, peritumoral, and combined texture features within and across the sequences, and evaluated their pCR prediction performance using AUC values. Results: The best single sequence model was SVM using a 1 mm tumor-to-peritumor VOI in the early contrast-enhanced phase (AUC = 0.9447). Among the combinations, the top-performing model was K-Nearest Neighbor, using 1 mm tumor-to-peritumor VOI in the early contrast-enhanced phase and 3 mm peritumoral VOI in T2FS (AUC = 0.9631). Conclusions: We suggest that a combined machine learning model that integrates tumoral and peritumoral radiomic features across different MRI sequences can provide a more accurate pretreatment pCR prediction for neoadjuvant chemotherapy in ER+HER2- LABC.

## 1. Introduction

Breast cancer is the most common female cancer worldwide [1,2]. Among the varied subtypes, ER+HER2- breast cancer has consistently increased in number since its incidence surpassed that of ER- breast cancer in 1950 [2]. Overall, ER+HER2- breast cancer has a good prognosis compared with other breast cancer subtypes. However, owing to its high incidence, it is the main subtype that accounts for the highest proportion of breast cancer mortality [2].

The treatment of ER+HER2- breast cancer can be broadly divided into early-stage breast cancer with no lymph node (LN) involvement and advanced-stage breast cancer with LN involvement. Currently, for early-stage breast cancer without LN involvement, the oncotype DX breast recurrence score is used as a quantitative measurement, using real-time PCR to predict the response to chemotherapy in patients requiring adjuvant chemotherapy [3]. For locally advanced breast cancer (LABC) with LN involvement, the standard protocol has so far been surgery following the completion of neoadjuvant chemotherapy (NAC) [4,5]. Regarding NAC, the lesion size is first reduced to improve operability, and notably, the pathologic complete response (pCR) upon operation after NAC has been proven to be a powerful prognostic factor for patients’ long-term outcomes [6,7,8].

Nonetheless, when the effect of NAC on ER+HER2- breast cancer is compared to that of other molecular subtypes of breast cancer, it is shown to have a poor NAC response [4,5]. According to a meta-analysis, the pCR rate of LABC upon the operation following NAC varied from 26.5% to 39.0% in other molecular subtypes, and the ER+HER2- subtype showed a significant difference, with a rate of 7.2% to 13.0% [9]. Given the significantly low NAC response in ER+HER2- LABC patients, it is crucial, from a precision medicine perspective, to selectively administer NAC to the approximately 10% of patients who exhibited a favorable response to NAC. This approach can help reduce unnecessary suffering for the 90% of patients who do not respond well to NAC, mitigating issues such as drug toxicity and delayed surgery. However, to date, there has been no reliable method for predicting this response, leading to uniform treatment strategies for all patients. Therefore, this study was focused on identifying, at an early stage, patients within the minority of patients with ER+HER2- LABC who exhibited a favorable response to NAC and who had the potential to achieve a pCR upon surgery.

We aimed to use magnetic resonance imaging (MRI), a representative modality for evaluating treatment response, to classify patients and potentially provide clinical assistance [6,7,10]. Initially, MRI assessed treatment response through lesion characteristics [11], but technological advances have expanded predictive techniques, such as multiparametric MRI, magnetic resonance spectroscopy (MRS), and FDG-PET [7,12]. Nevertheless, designing accurate and reproducible parameters remains a challenge. Recent research has actively explored MRI radiomics, utilizing texture features [3,10]. MRI texture analysis (TA) offers objective assessment by quantifying data representing tissue heterogeneity, often imperceptible visually [13]. This has led to efforts to enhance reproducibility through machine learning models trained using a selected set of key texture features [14].

Most previous studies that used MRI to predict pCR rates among patients with breast cancer can be broadly divided into two general aspects based on the region of interest in the imaging. First, many studies have attempted to explain treatment responses based on temporal changes in lesions between the initial MRI and the early or mid-term MRI after NAC [15,16]. However, such a prediction based on a comparison between the initial lesion and the residual lesion in a follow-up MRI has been reported to over- or under-estimate the lesion due to various changes associated with the treatment response [17]. More importantly, decreased quality of life experienced by patients who receive unnecessary treatment or have delayed appropriate treatment should be considered. Thus, this study focused on refining the prediction of pCR using pretreatment MRI. Second, in many previous studies, when evaluating lesions on MRI, the focus was mainly on the tumor region [18,19,20]; however, in this study, the peritumoral region was also evaluated and analyzed. Several studies have shown that the peritumoral region can be critical to the response to NAC by reflecting angiogenic or lymphangiogenic activity [21,22]. This study focused more on confirming the importance of the peritumoral region. Thus far, a few recent studies have attempted to use pretreatment MRI only or have included the peritumoral region as a consideration [21,23]. However, these studies considered the ER+HER2- subtype, the focus of this study, only as a part of the study population, and no study has yet investigated the pretreatment NAC response with a focus on the ER+HER2- subtype.

Therefore, this study aimed to develop and validate a reproducible practical machine learning model with texture features incorporating both tumoral and peritumoral regions across initial pretreatment MRI sequences in patients with ER+HER2- LABC, whose NAC response was notably low. Through this study, we hope to provide practical help to clinicians in establishing tailored therapeutic strategies by stratifying this patient population prior to treatment.

## 2. Materials and Methods

### 2.1. Patient Population and Study Design

This retrospective study was approved by the institutional review board of our hospital, which waived the requirement for informed consent.

Between January 2010 and December 2020, 2349 patients with advanced breast cancer received NAC at our institution. Among these, 818 were diagnosed with ER+HER2- LABC subtype. First, 403 patients were excluded because of a lack of raw data for the dynamic study in the Picture Archiving and Communication System (PACS). Patients with a history of previous treatment, no cytology report on the initial axillary LN metastasis, no report on the final pathological results, no pretreatment MRI, no verifiable information on the four MRI sequences essential to this study (T1-weighted fat-suppressed pre-contrast, early and delayed post-contrast subtraction sequences, and T2-weighted fat-suppressed sequence), and insufficient image quality for lesion segmentation were excluded. Finally, the inclusion criteria were patients who (1) had a pretreatment MRI performed at our center, (2) completed all cycles of NAC and had surgery with a final pathologic report on achievement of pCR or not, and (3) had all four sequences with sufficient quality for segmentation, resulting in a total of 265 enrolled patients (stage IIB through IIIC according to the 8th edition of the AJCC cancer staging system).

**Figure 1:**
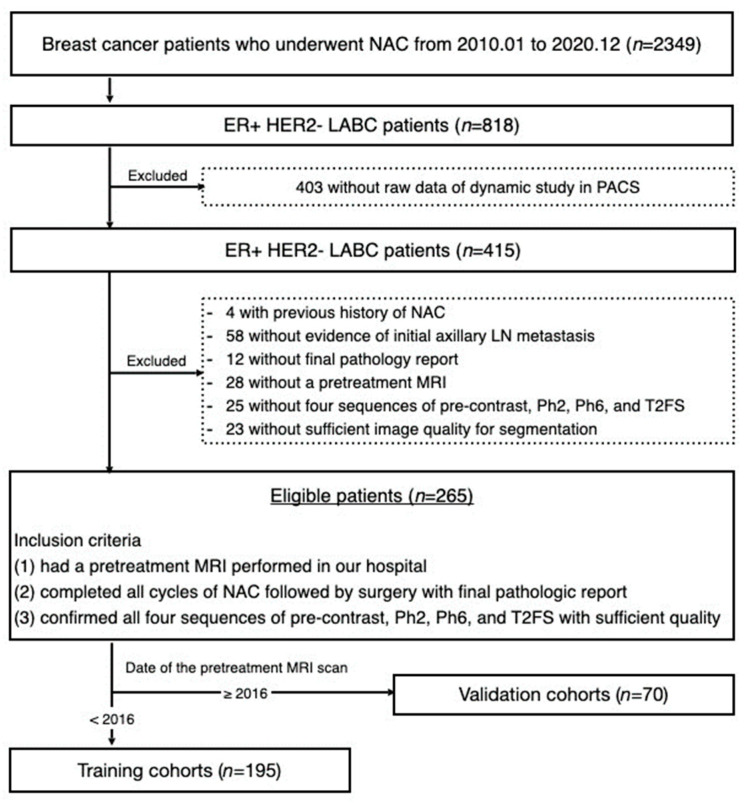
Flowchart of patient selection and data set. Abbreviations: NAC—neoadjuvant chemotherapy; LABC—locally advanced breast cancer; PACS—Picture Archiving and Communication System; LN—lymph node; MRI—magnetic resonance imaging; Ph2—T1-weighted fat-suppressed early post-contrast subtraction sequences; Ph6—T1-weighted fat-suppressed delayed post-contrast subtraction sequences; T2FS—T2-weighted fat-suppressed sequence.

Based on the date of the pretreatment MRI scans, patients were divided into training and validation cohorts. A total of 195 patients who underwent MRI between 2010 and 2015 were included in the training cohort. Another 70 patients who underwent MRI between 2016 and 2020 were included in the temporal validation cohort. The patient selection process is illustrated in Figure 1.

### 2.2. MRI Acquisition

Breast MRI examinations were performed with the patients in the prone position using a 3.0 T scanner (MR750, GE Healthcare, Milwaukee, WI, USA or TrioTim, Siemens Healthcare, Erlangen, Germany using a dedicated eight- or four-channel breast coil). The following images have been commonly obtained after the localizer images from one of the two types of scanners: T2-weighted fast spin echo axial images (TR/TE, 9100/100 ms; flip angle, 110°; matrix, 416 × 256 pixels; section thickness, 3 mm, or TR/TE, 4300/80 ms; flip angle, 150°; matrix, 512 × 512 pixels; section thickness, 3 mm), T2-weighted short-time inversion recovery (STIR) axial images (TR/TE, 5000/70 ms; inversion time, 200 ms; flip angle, 110°; matrix, 320 × 256 pixels; section thickness, 3 mm), and T1-weighted fat-suppressed pre-contrast and 3D dynamic post-contrast enhanced (DCE) axial images (TR/TE, 5.6/1.7 ms; flip angle, 12°; matrix, 280 × 512 pixels; section thickness, 3 mm, or TR/TE, 280/2.6 ms; flip angle, 65°; matrix, 343 × 512 pixels; section thickness, 3 mm) with one pre-contrast and six post-contrast dynamic series obtained before and after a bolus injection of 0.1 mmol/kg body weight of gadolinium-based contrast agent (Dotarem, Guerbet, Paris, France; Magnevist, Berlex Laboratories, Wayne, NJ, USA, or Gadovist, Bayer Schering Pharma, AG, Berlin, Germany) at a rate of 2 mL/s, followed by 20 mL saline flush. Post-processing, image subtraction was performed by subtracting pre-contrast images from the post-contrast images. The field of view was 32–34 cm for all MRI sequences.

### 2.3. Volume of Interest (VOI) Segmentation

**Figure 2:**
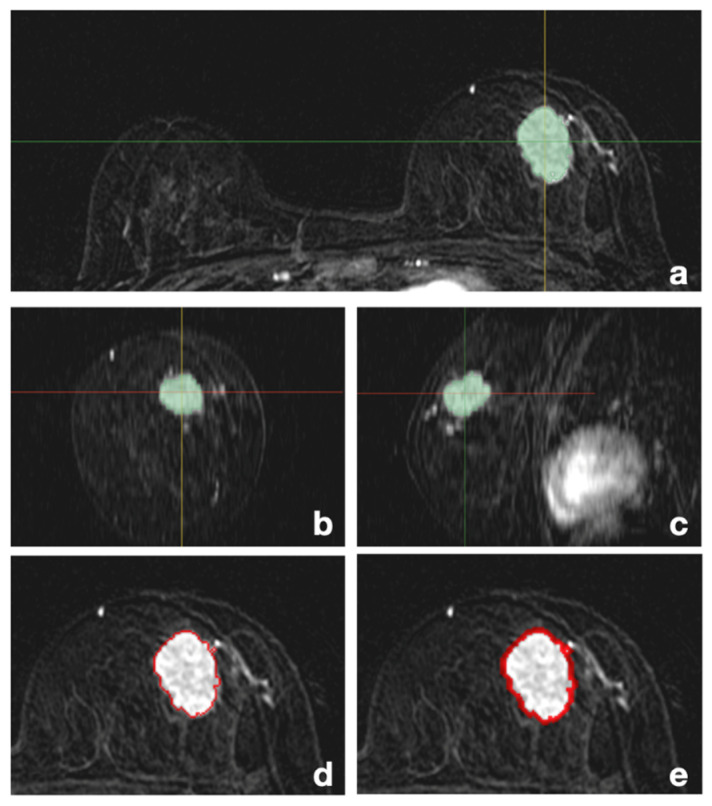
Segmentation on T1-weighted fat-suppressed early post-contrast subtraction sequence for a patient with histologically confirmed ER+HER2- LABC: (**a**) axial tumoral masks; (**b**) coronal tumoral masks; (**c**) sagittal tumoral masks; (**d**) axial-1 mm peritumoral mask; (**e**) axial-3 mm peritumoral mask. Abbreviations: LABC—locally advanced breast cancer.

The VOI segmentation of tumors was first semi-automatically performed along the margin of the tumor in the axial scan of T1-weighted fat-suppressed early post-contrast subtraction sequences (Ph2) by a radiologist (J.P., with 5 years of experience in radiology) using 3D-Slicer (version 5.0.2) software, and the accuracy of the image up to the 3D margin on the coronal and sagittal planes was checked with necessary modifications. For peritumoral VOI segmentation, the existing tumor mask was subtracted after 3D dilation by 1 mm and 3 mm units (Figure 2).

The same process was applied to T1-weighted fat-suppressed delayed post-contrast subtraction sequences (Ph6) and T2-weighted fat-suppressed sequences (T2FS). Thus, 15 VOIs of tumoral, peritumoral (1 mm, 3 mm), and tumoral + peritumoral (1 mm, 3 mm) were obtained for Ph2, Ph6, and T2FS in each patient’s pretreatment MRI. The process was evaluated by another senior radiologist (M.J.K., with 23 years of experience in radiology) to assess and revise the tumoral and peritumoral VOI segmentations to reconfirm the entire procedure.

### 2.4. MRI Preprocessing and Radiomic Texture Feature Extraction

For the segmented VOIs, N4ITK MRI bias correction was applied to improve the non-uniformity of MR images between different patients [24], and the variation between data was minimized by normalizing the gray-level value, as shown in the following formula [25]:

$$f\left(x\right)=\frac{s(x-\mu_{x})}{\sigma_{x}}$$

Here, x is the amplitude of the image, $\mu_{x}$ is the average of the image values, $\sigma_{x}$ is the standard deviation of the image, and s is an optional scaling value set to 10 to prevent errors in the calculation of radiomic features that may occur due to a relatively large standard deviation. After resampling the image with a 1 × 1 × 1 mm iso-voxel, 863 radiomic features were extracted from each VOI in the three sequences. Among the extracted features, diagnostic features (*n* = 12), which are information on the entire image, not VOI, and shape features among the original features (*n* = 14), which were information related to tumor size or volume measurable on conventional MRI, were excluded. The final feature set incorporated 2511 features for each sequence, and 7533 features were extracted from each patient.

### 2.5. Dimension Reduction

Python 3.8 was used for data handling in the machine learning steps, and the key feature selection on the radiomic features extracted from each VOI was performed in two steps. First, the Mann–Whitney U test was used with statistical significance related to pCR or non-pCR prediction (*p* < 0.05). Second, using the random forest (RF) algorithm, the top 30 features were selected for radiomic feature importance in pCR prediction. Prior to data training, a standard scaler was applied to adjust the deviating scales of the radiomic features and reduce the influence of outliers. Additionally, the synthetic minority over-sampling technique (SMOTE) was performed to reduce the problem of overfitting toward non-pCR due to the numerical imbalance between the pCR and non-pCR groups, even if the number reflected the actual clinical pCR rate of ER+HER2- LABC.

### 2.6. Development of pCR Prediction Model in the Training Cohort

First, pCR prediction model development was individually developed for each sequence. Seven representative machine learning models were created with the key radiomic features for each of the five VOIs (tumor, peritumor 1 mm, peritumor 3 mm, area from tumor to peritumor 1 mm, and area from tumor to peritumor 3 mm) in the MRI sequences of the training cohort: binary classification model, K-Nearest Neighbor model, Support Vector Machine (SVM), Decision Tree classifier, AdaBoost classifier, Random Forest (RF) classifier, and Light Gradient-Boosting Machine (LightGBM). A grid search approach was used to find the best hyperparameters for each of these seven models. This method systematically explores various combinations of hyperparameters to identify the optimal configuration for each model. To evaluate the performance of each model and its hyperparameter combination, we employed a five-fold cross-validation. The dataset was divided into five subsets. During each iteration, four subsets were designated for training, while the remaining subset was allocated for validation. This cycle was repeated five times, ensuring that each subset served as the validation set once. The optimal model and its hyperparameters for each VOI were selected based on the area under the curve (AUC) value, representing the true positive rate (sensitivity) plotted against the false positive rate (1-specificity) [26], which measures the model’s ability to differentiate between the pCR and non-pCR groups.

Next, to construct a more sophisticated pCR prediction model, seven machine learning models were created with sets of selected key radiomic features in combination for tumoral, peritumoral, and tumoral + peritumoral VOIs across sequences from the training cohort. The training and testing processes were identical to those described as above, and AUC values were used to select the optimal model.

Finally, a model incorporating clinical factors instead of radiomic features was created as a comparison group, and its performance was evaluated. Excluding the molecular subtype and axillary LN metastasis of breast cancer, which were fixed in this study, patient age, tumor size, and estrogen receptor (ER) and progesterone receptor (PR) expression levels were selected as clinical factors potentially associated with disease prognosis.

### 2.7. Assessment of pCR Prediction Model Performance with the Validation Cohort

We validated the predictive performance of the optimal models developed using radiomic features extracted from the VOIs of each sequence, radiomic features combined from the VOIs across different sequences, and clinical factors in the validation cohort. After calculating the AUC, precision (the ratio of correctly predicted positive observations to the total predicted positives), recall (the ratio of correctly predicted positive observations to total actual positives), and F1 scores (the harmonic mean of Precision and Recall, representing both precision and recall in one metric), the predictive performance of the model was evaluated using the AUC of the receiver operating characteristic (ROC) curve [27].

$$\mathrm{Precision}=\frac{\mathrm{True}\;\mathrm{Positive}}{\mathrm{True}\;\mathrm{Positive}+\mathrm{False}\;\mathrm{Positive}}$$

$$\mathrm{Recall}=\frac{\mathrm{True}\;\mathrm{Positive}}{\mathrm{True}\;\mathrm{Positive}+\mathrm{False}\;\mathrm{Negative}}$$

$$F1\;\mathrm{Score}=2\times \frac{\mathrm{Precision}\times \mathrm{Recall}}{\mathrm{Precision}+\mathrm{Recall}}$$

**Figure 3:**
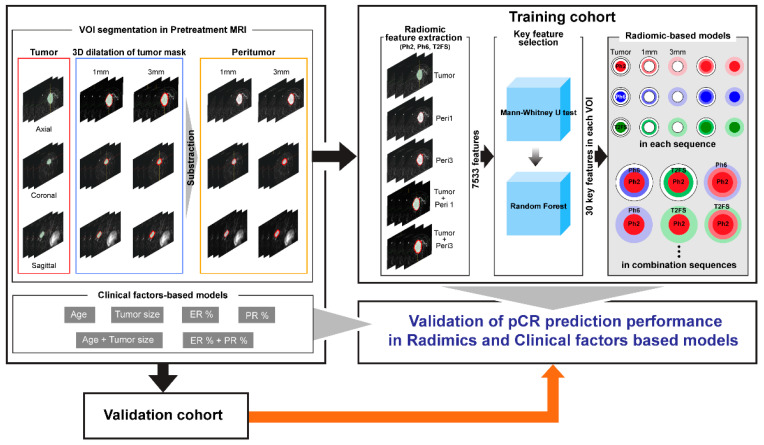
Radiomics workflow used in this study. Abbreviations: Ph2—T1-weighted fat-suppressed early post-contrast subtraction sequence; Ph6—T1-weighted fat-suppressed delayed post-contrast subtraction sequences; T2FS—T2-weighted fat-suppressed sequence.

The process of this study is summarized in Figure 3.

### 2.8. Statistical Analysis

Statistical analyses of clinical factors, including patient age, tumor size, and ER and PR expression levels, were performed as follows: continuous variables were expressed as means and standard deviations, while categorical variables were expressed as frequencies and percentages. Continuous variables were tested using the independent samples t-test or the Mann–Whitney U test based on the results of the Shapiro–Wilk test for normality, and categorical variables were compared using the χ^2^ test. Statistical significance was accepted when *p* values were <0.05.

## 3. Results

### 3.1. Patient Characteristics

This study included pretreatment MRIs scans of 265 patients with ER+HER2- LABC with axillary LN metastasis. The clinical and histological factors of the pCR and non-pCR groups, considering pCR as the endpoint in this study, are shown in Table 1. Among the patients, 238 (89.8%) had a non-pCR and 27 (10.2%) reached pCR. The mean tumor size between the pCR and non-pCR groups was observed to be significantly different, with 37.9 ± 21.3 mm and 22.1 ± 8.8 mm, respectively. However, there were no significant differences in terms of patient age and estrogen and progesterone receptor expression levels between the pCR and non-pCR groups.

A comparison of the training and validation cohorts is shown in Table 2. The two cohorts showed no significant difference in pCR rate (9.7% and 11.4%, respectively). Table 2 shows the other characteristics of the two cohorts, including patient age, tumor size, and estrogen and progesterone receptor expression levels.

### 3.2. Radiomic Texture Feature Composition and Dimension Reduction

As previously mentioned, excluding diagnostic and shape features, 837 radiomic texture features per VOI were extracted from each patient’s pretreatment MRI. The 837 radiomic texture features included 93 original (first-order, shape, gray-level co-occurrence matrix [GLCM], gray-level dependence matrix [GLDM], gray-level run-length matrix [GLRLM], gray-level size-zone matrix [GLSZM], and neighboring gray tone difference matrix [NGTDM]), and 744 wavelet features (Table S1). The Mann–Whitney U test was used to remove 16 features showing no significant difference between pCR and non-pCR. The remaining 821 features were ranked by importance values from the Random Forest (RF) algorithm, and the top 30 features were chosen.

### 3.3. Performance of the pCR Prediction Model in Each Sequence

**Figure 4:**
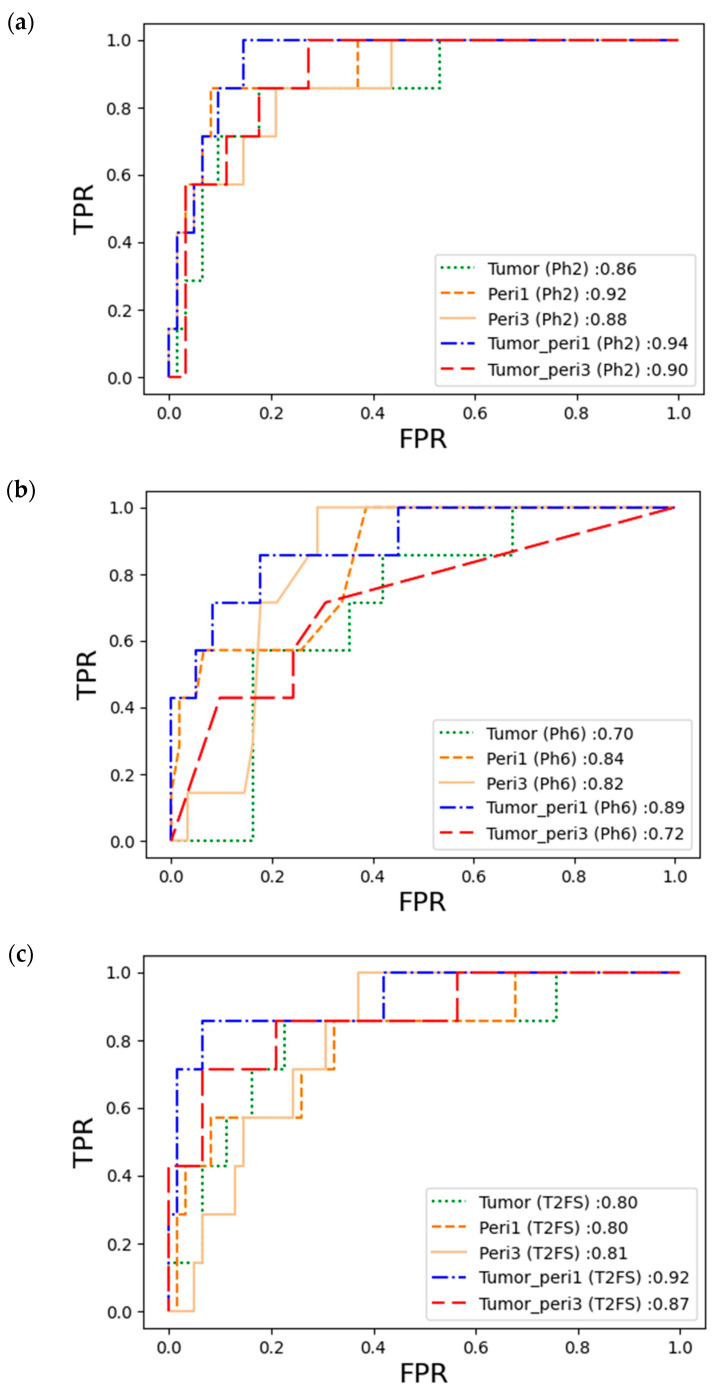
The ROC curve for the predictive performance of pCR in the validation cohort using the optimal machine learning models in each sequence: (**a**) AUC on Ph2; (**b**) AUC on Ph6; (**c**) AUC on T2FS. Abbreviations: Ph2—T1-weighted fat-suppressed early post-contrast subtraction sequence; Ph6—T1-weighted fat-suppressed delayed post-contrast subtraction sequence; T2FS—T2-weighted fat-suppressed sequence; Peri1—peritumoral region, 1 mm; Peri3—peritumoral region, 3 mm; Tumor_peri1—tumoral + 1 mm peritumoral region; Tumor_peri3—tumoral + 3 mm peritumoral region; FPR—false positive rate; TPR—true positive rate.

Table S2 presents the final pCR prediction performance in the validation cohort, which was confirmed by sequentially applying the optimal machine learning models developed from each of the five types of VOIs. A general look at the table reveals that the models derived from Ph2 and T2FS showed relatively high AUC values, whereas even the best performing models in Ph6 did not exceed an AUC value of 0.9. The best model for pCR prediction of NAC in ER+HER2- LABC in the three respective sequences was the SVM model of tumor-to-peritumor 1 mm on Ph2 (AUC = 0.9447, recall = 91%, precision = 91%, and F1 score = 91%). The ROC curves and AUCs of the 15 models in the validation cohorts are shown in Figure 4, and it can be confirmed once again that the overall high AUC value was shown in Ph2.

### 3.4. Performance of the pCR Prediction Model with Combination of Sequences

**Figure 5:**
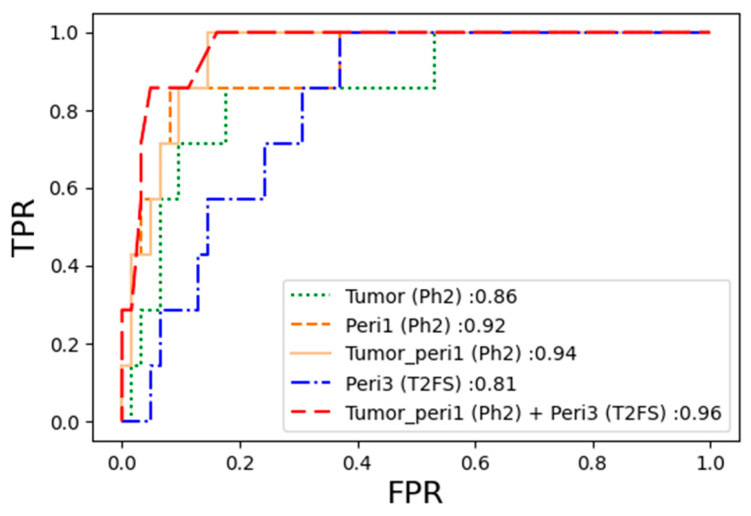
Comparison of the pCR prediction performance in the validation cohort: The best combination model of the VOI from tumor-to-peritumor 1 mm in Ph2 and the peritumor 3 mm VOI in T2FS as well as the respective components of the VOI models. Abbreviations: Ph2—T1-weighted fat-suppressed early post-contrast subtraction sequence; T2FS—T2-weighted fat-suppressed sequence; Peri1—peritumoral region, 1 mm; Tumor_peri1—tumoral + 1 mm peritumoral region; Peri3—peritumoral region, 3 mm; FPR—false positive rate; TPR—true positive rate.

We confirmed the predictive performance of pCR for the optimal machine learning model developed from 75 VOIs, combining the tumoral and peritumoral regions in two different sequences in the validation cohort. The KNN model with key radiomic features derived from a combination of VOIs ranging from the tumor-to-peritumor 1 mm in Ph2 and peritumor 3 mm VOI in T2FS exhibited the best pCR prediction performance, with an AUC of 0.96. The pCR prediction performance based on the combination of tumoral and peritumoral regions of different sequences is shown in Table S3. Additionally, Figure 5 compares the ROC curve of the optimal model developed using the tumoral VOI, peritumoral 1 mm VOI, tumor-to-peritumoral 1 mm VOI of Ph2, and peritumoral 3 mm VOI of T2FS, which are components of the combination model.

Cochran’s Q test verified that there was a significant difference between these five models (*p* < 0.001).

### 3.5. Diagnostic Performance of Clinical Model

Furthermore, we used the same process to confirm the predictive performance of pCR for clinical factors that could be associated with patient prognosis in breast cancer, such as age, tumor size, and estrogen and progesterone expression levels.

**Figure 6:**
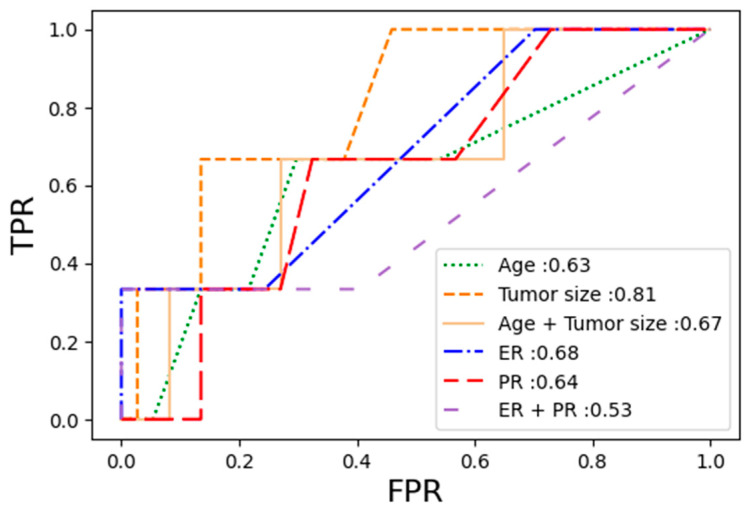
The ROC curve for pCR prediction performances of the clinical models in the validation cohort. Abbreviations: ER—estrogen; PR—progesterone; FPR—false positive rate; TPR—true positive rate.

In the validation cohort, the AUC values were generally low for pCR prediction performance compared to the radiomics models. The AUC values for patient age, tumor size, and the combination model of patient age and tumor size were 0.63, 0.81, and 0.67, respectively. The AUC values for estrogen and progesterone expression levels and their combination model were 0.68, 0.64, and 0.53, respectively. The results are summarized in Table S4 and Figure 6.

## 4. Discussion

ER+HER2- locally advanced breast cancer (LABC) has a poor pathologic complete response (pCR) rate of approximately 10% compared with the 3–40% pCR rates of other molecular subtypes after surgical intervention following neoadjuvant chemotherapy (NAC) [9]. Therefore, this study aimed to classify ER+HER2- LABC patients with a high probability of providing an effective response to NAC using pretreatment MRI, which is a key modality for the non-invasive assessment of breast cancer [6,7,10]. Several recent studies have attempted to create a prognosis prediction model for breast cancer using radiomic texture feature extraction with respect to the pretreatment MRI applied in this study [10,21,28]. However, all these studies were conducted on heterogeneous molecular subtypes with only a small part of the patient population with ER+HER2- LABC, which is the focus of this study.

To construct a sophisticated model for pCR prediction after NAC in patients with ER+HER2- LABC, the radiomic texture features of MRI were extracted from the tumor, peritumor 1 mm, peritumor 3 mm, area from tumor-to-peritumor 1 mm, and area from tumor-to-peritumor 3 mm for early post-contrast sequences, delayed post-contrast sequences, and T2-weighted fat-saturated sequences. In line with previous studies, it was further established that early post-contrast images predominantly contain the most useful texture features in machine learning models as a single sequence model evaluation [29,30,31]. The inclusion of the delayed post-contrast image in this study was based on a previous study by Jin et al., who claimed that texture heterogeneity is better reflected in the delayed enhanced phase for breast tumors [32]. However, the model incorporating the texture features of the tumor in the delayed phase did not produce more potent information in comparison to other sequences in our study.

Another strength of this study is that not only is the tumoral region the basis for determining the VOI for radiomic feature extraction in MRI, but the peritumoral region, which is also reported to form a microenvironment that affects the NAC response [21,22], was included. Until recently, studies included the peritumoral region to investigate an extended area from the tumoral to the peritumoral region on a single MRI sequence [22]. In this study, on the other hand, the tumoral and peritumoral regions in combination across sequences were examined to construct a more advanced model that reflects more important sequences related to each region. As a result, the model with a combination of the extended tumoral region in the early enhanced phase and the peritumoral region in T2FS exhibited the highest AUC. This finding is consistent with the general MRI principle that T2FS images exhibit a wider range of signal alterations compared to T1-weighted images, even including contrast-enhanced T1-weighted images [33,34]. In the future, more elaborate models need to be developed by combining tumoral and peritumoral regions across different sequences and validated for other molecular subtypes of breast cancer.

This study had several limitations. First, there was a possibility of selection bias because the study was a retrospective study conducted at a single tertiary referral center, and due to the unavailability of raw data for MRI dynamic studies from patients accumulated over a substantial ten-year period, certain patients had to be excluded. Second, this study intentionally focused on ER+HER2- LABC, which is a molecular subtype with relatively poor NAC response. Because the results were based on a single molecular subtype, it may be difficult to generalize the findings to all patients with breast cancer. We hope that follow-up studies will be conducted in future. Third, regarding the potential clinical utility, more time seems necessary for the immediate clinical application of the findings through rapid and reliable automatic segmentation. An accurate VOI segmentation process for a tumor is a prerequisite for providing accurate key texture features to constitute machine learning models. Although this study used a 3D slicer to produce images in a semi-automatic manner, the reliability of the VOI produced by the program decreased as the irregularity of the tumor margin increased, which required modification by a radiologist and reconfirmation by a senior radiologist to refine the VOI segmentation. Lastly, the most fundamental limitation was found in the revision of treatment plans for patients with ER+HER2- LABC. Despite a mere 10% pCR rate after NAC, NAC is still administered to patients with ER+HER2- LABC, mainly because more effective and specific treatments for this patient group are still in progress. However, if we accumulate evidence supporting the accurate classification of patients who exhibited a favorable response to NAC before treatment initiation, we can significantly influence practical treatment decisions made by oncologists and surgeons by instilling confidence grounded in sound reasoning. This approach could potentially shift the focus for ER+HER2- LABC patients expected to exhibit a poor response to NAC towards earlier surgical interventions and the determination of the scope of post-surgery treatment, leveraging tools such as Oncotype Dx for adjuvant chemotherapy decisions. Consequently, we anticipate that these efforts will assist in the establishment of tailored therapy plans that prioritize benefits over risks, ultimately improving patient quality of life.

To assess the NAC response of patients with ER+HER2- LABC on pretreatment MRI, this study applied radiomic texture features to the tumoral and peritumoral regions across MRI sequences. We suggest that a combination of machine learning models incorporating tumoral and peritumoral texture features across different MRI sequences can provide a more accurate prediction of pCR for NAC response in these patients. These results are also expected to make a potential contribution to the development of novel clinical therapeutic strategies.

## Figures and Tables

**Table 1:** Comparison of the patient characteristics between non-pCR and pCR groups.

	Non-pCR	pCR	*p* Value
	*n* = 238 (89.8%)	*n* = 27 (10.2%)	
Age, years	49.2 ± 9.1	48.6 ± 6.2	0.975
Tumor size, mm	37.9 ± 21.3	22.1 ± 8.8	<0.001
ER expression, %	85.6 ± 20.5	75.3 ± 30.7	0.058
PR expression, %	34.3 ± 36.3	28.9 ± 38.9	0.386

Abbreviations: pCR—pathologic complete response; ER—estrogen receptor; PR—progesterone receptor.

**Table 2:** Comparison of the patient characteristics between the training and validation cohorts.

		Train	Validation	*p* Value
		*n* = 195 (73.6%)	*n* = 70 (26.4%)	
Pathology	non-pCR	176 (90.3%)	62 (88.6%)	0.865
	pCR	19 (9.7%)	8 (11.4%)	
Age, years		48.5 ± 8.4	51.0 ± 9.9	0.043
Tumor size, mm		35.0 ± 21.1	40.0 ± 19.9	0.086
ER expression, %		85.2 ± 21.7	82.8 ± 22.5	0.408
PR expression, %		30.9 ± 35.7	41.7 ± 38.0	0.016

Abbreviations: pCR—pathologic complete response; ER—estrogen receptor; PR—progesterone receptor.

## Data Availability

Most of the research data is detailed in the Supplementary Materials. Additionally, we are limiting data access for other research data due to privacy and ethical constraints.

## Supplementary Materials

The following supporting information can be downloaded at: https://www.mdpi.com/article/10.3390/diagnostics13193031/s1, Table S1: The radiomic features extracted using 3D Slicer PyRadiomics; Table S2: The predictive performance of pCR in the validation cohort using the optimal machine learning models developed for tumoral, peritumoral, and tumoral + peritumoral VOIs in each sequence; Table S3: Predictive performance of pCR in the validation cohort using the optimal machine learning models based on combination of tumoral and peritumoral regions across sequences; Table S4: pCR prediction performances of the clinical models in the validation cohort.
