# Supplementary material for: Machine Learning Predicts Pathologic Complete Response to Neoadjuvant Chemotherapy for ER+HER2- Breast Cancer: Integrating Tumoral and Peritumoral MRI Radiomic Features
Source: Diagnostics (Basel). 2023 Sep 23;13(19):3031. doi: 10.3390/diagnostics13193031 (PMC10572844; doi:10.3390/diagnostics13193031)
Supplement: Supplementary file 1 [file diagnostics-13-03031-s001.zip › diagnostics-2622680-supplementary.pdf]

Table S1. The radiomic features extracted using 3D Slicer PyRadiomics

Image type	Feature Class	Num of features
original	First-order	18
original	GLCM	24
original	GLDM	14
original	GLRLM	16
original	GLSZM	16
original	NGTDM	5
wavelet-HHH	First-order	18
wavelet-HHH	GLCM	24
wavelet-HHH	GLDM	14
wavelet-HHH	GLRLM	16
wavelet-HHH	GLSZM	16
wavelet-HHH	NGTDM	5
wavelet-HHL	First-order	18
wavelet-HHL	GLCM	24
wavelet-HHL	GLDM	14
wavelet-HHL	GLRLM	16
wavelet-HHL	GLSZM	16
wavelet-HHL	NGTDM	5
wavelet-HLH	First-order	18
wavelet-HLH	GLCM	24
wavelet-HLH	GLDM	14
wavelet-HLH	GLRLM	16
wavelet-HLH	GLSZM	16

wavelet-HLH	NGTDM	5
wavelet-HLL	First-order	18
wavelet-HLL	GLCM	24
wavelet-HLL	GLDM	14
wavelet-HLL	GLRLM	16
wavelet-HLL	GLSZM	16
wavelet-HLL	NGTDM	5
wavelet-LHH	First-order	18
wavelet-LHH	GLCM	24
wavelet-LHH	GLDM	14
wavelet-LHH	GLRLM	16
wavelet-LHH	GLSZM	16
wavelet-LHH	NGTDM	5
wavelet-LHL	First-order	18
wavelet-LHL	GLCM	24
wavelet-LHL	GLDM	14
wavelet-LHL	GLRLM	16
wavelet-LHL	GLSZM	16
wavelet-LHL	NGTDM	5
wavelet-LLH	First-order	18
wavelet-LLH	GLCM	24
wavelet-LLH	GLDM	14
wavelet-LLH	GLRLM	16
wavelet-LLH	GLSZM	16
wavelet-LLH	NGTDM	5

wavelet-LLL	First-order	18
wavelet-LLL	GLCM	24
wavelet-LLL	GLDM	14
wavelet-LLL	GLRLM	16
wavelet-LLL	GLSZM	16
wavelet-LLL	NGTDM	5

Abbreviations: GLCM, gray-level co-occurrence matrix; GLDM, gray-level dependence matrix; GLRLM, gray-level run-length matrix; GLSZM, gray-level size- zone matrix; NGTDM, neighboring gray tone difference matrix.

Table S2. The predictive performance of pCR in the validation cohort using the optimal machine learning models developed for tumoral, peritumoral, and tumoral + peritumoral VOIs in each sequence

Type	Rank	AUC	Precision	Recall	F1-score	Best Model
Tumor (Ph2)	8	0.8594	0.9093	0.8696	0.8840	SVC
Tumor (Ph6)	15	0.7005	0.8731	0.7826	0.8166	Random Forest
Tumor (T2FS)	12	0.8018	0.8984	0.6667	0.7327	SVC
Peri1 (Ph2)	3	0.9171	0.9335	0.8986	0.9098	Logistic Regression
Peri1 (Ph6)	9	0.8422	0.9051	0.8986	0.9015	K Neighbors
Peri1 (T2FS)	13	0.7995	0.8851	0.8406	0.8582	Random Forest
Peri3 (Ph2)	6	0.8756	0.8934	0.8696	0.8795	Random Forest
Peri3 (Ph6)	10	0.8249	0.8366	0.7826	0.8066	AdaBoost Classifier
Peri3 (T2FS)	11	0.8134	0.9215	0.6522	0.7203	Logistic Regression
Tumor_peri1 (Ph2)	1	0.9447	0.9130	0.9130	0.9130	SVC
Tumor_peri1 (Ph6)	5	0.8917	0.9193	0.9275	0.9185	AdaBoost Classifier
Tumor_peri1 (T2FS)	2	0.9240	0.9442	0.9275	0.9330	AdaBoost Classifier

Tumor_peri3 (Ph2)	4	0.9009	0.8061	0.8841	0.8433	Random Forest
Tumor_peri3 (Ph6)	14	0.7189	0.8450	0.7246	0.7718	Decision Tree Classifier
Tumor_peri3 (T2FS)	7	0.8710	0.9193	0.9275	0.9185	SVC

Abbreviations: Ph2, T1-weighted fat-suppressed early post-contrast subtraction sequence; Ph6, T1-weighted fat-suppressed delayed post-contrast subtraction sequences; T2FS, T2-weighted fat-suppressed sequence, Peri1, peritumoral region, 1mm; Peri3, peritumoral region, 3mm Tumor_peri1, tumoral + 1mm peritumoral region; Tumor_peri3, tumoral + 3mm peritumoral region; SVM, Support Vector Machine.

Table S3. Predictive performance of pCR in the validation cohort using the optimal machine learning models based on combination of tumoral and peritumoral regions across sequences

Type1	Type2	Rank	AUC	Precision	Recall	F1-score	Best Model
Tumor_peri1 (Ph2)	Tumor (Ph6)	5	0.9378	0.9207	0.9130	0.8824	SVC
Tumor_peri1 (Ph2)	Tumor (T2FS)	9	0.9194	0.9335	0.8986	0.9098	SVC
Tumor_peri1 (Ph2)	Peri1 (Ph6)	6	0.9309	0.9329	0.9275	0.9088	AdaBoost
Tumor_peri1 (Ph2)	Peri1 (T2FS)	21	0.9032	0.9196	0.8406	0.8655	SVC
Tumor_peri1 (Ph2)	Peri3 (Ph6)	9	0.9194	0.8981	0.9130	0.8970	LGBM
Tumor_peri1 (Ph2)	Peri3 (T2FS)	1	0.9631	0.9234	0.9275	0.9250	K Neighbors
Tumor_peri1 (Ph2)	Tumor_peri1 (Ph6)	14	0.9101	0.8981	0.9130	0.8970	LGBM
Tumor_peri1 (Ph2)	Tumor_peri1 (T2FS)	12	0.9171	0.9137	0.8841	0.8950	SVC
Tumor_peri1 (Ph2)	Tumor_peri3 (Ph6)	6	0.9309	0.9234	0.9275	0.9250	LGBM
Tumor_peri1 (Ph2)	Tumor_peri3 (T2FS)	2	0.9505	0.9325	0.9275	0.9297	K Neighbors

Tumor_peri1 (T2FS)	Tumor (Ph2)	25	0.8963	0.8987	0.8841	0.8904	LGBM
Tumor_peri1 (T2FS)	Tumor (Ph6)	51	0.8594	0.9093	0.8696	0.8840	AdaBoost
Tumor_peri1 (T2FS)	Peri1 (Ph2)	9	0.9194	0.9420	0.9420	0.9420	Logistic
Tumor_peri1 (T2FS)	Peri1 (Ph6)	28	0.8940	0.9188	0.8986	0.9062	LGBM
Tumor_peri1 (T2FS)	Peri3 (Ph2)	17	0.9078	0.9420	0.9420	0.9420	LGBM
Tumor_peri1 (T2FS)	Peri3 (Ph6)	55	0.8525	0.9021	0.8406	0.8624	Logistic
Tumor_peri1 (T2FS)	Tumor_peri1 (Ph6)	41	0.8779	0.8963	0.8116	0.8411	AdaBoost
Tumor_peri1 (T2FS)	Tumor_peri3 (Ph2)	14	0.9101	0.8981	0.9130	0.8970	Random Forest
Tumor_peri1 (T2FS)	Tumor_peri3 (Ph6)	58	0.8364	0.8816	0.8261	0.8478	Logistic
Peri1 (Ph2)	Tumor (Ph6)	31	0.8894	0.9033	0.9130	0.9065	LGBM
Peri1 (Ph2)	Tumor (T2FS)	19	0.9055	0.9188	0.8986	0.9062	Logistic
Peri1 (Ph2)	Peri1 (Ph6)	38	0.8802	0.9021	0.8406	0.8624	Logistic
Peri1 (Ph2)	Peri1 (T2FS)	32	0.8871	0.9130	0.9130	0.9130	K Neighbors
Peri1 (Ph2)	Peri3 (Ph6)	43	0.8733	0.8922	0.8986	0.8951	Random Forest
Peri1 (Ph2)	Peri3 (T2FS)	17	0.9078	0.9130	0.9130	0.9130	Logistic
Peri1 (Ph2)	Tumor_peri1 (Ph6)	48	0.8641	0.9455	0.9420	0.9313	LGBM
Peri1 (Ph2)	Tumor_peri3 (Ph6)	32	0.8871	0.9130	0.9130	0.9130	Logistic

Peri1 (Ph2)	Tumor_peri3 (T2FS)	30	0.8917	0.9420	0.9420	0.9420	Random Forest
Tumor_peri3 (Ph2)	Tumor (Ph6)	71	0.7926	0.8032	0.8551	0.8284	Random Forest
Tumor_peri3 (Ph2)	Tumor (T2FS)	43	0.8733	0.9051	0.8986	0.9015	Random Forest
Tumor_peri3 (Ph2)	Peri1 (Ph6)	3	0.9401	0.9193	0.9275	0.9185	LGBM
Tumor_peri3 (Ph2)	Peri1 (T2FS)	56	0.8433	0.9033	0.9130	0.9065	Random Forest
Tumor_peri3 (Ph2)	Peri3 (Ph6)	21	0.9032	0.8507	0.8841	0.8627	Random Forest
Tumor_peri3 (Ph2)	Peri3 (T2FS)	38	0.8802	0.8346	0.8551	0.8441	LGBM
Tumor_peri3 (Ph2)	Tumor_peri1 (Ph6)	19	0.9055	0.8802	0.8986	0.8859	LGBM
Tumor_peri3 (Ph2)	Tumor_peri3 (Ph6)	43	0.8733	0.8611	0.8696	0.8651	Random Forest
Tumor_peri3 (Ph2)	Tumor_peri3 (T2FS)	8	0.9274	0.9137	0.8841	0.8950	K Neighbors
Tumor_peri1 (Ph6)	Tumor (Ph2)	12	0.9171	0.8074	0.8986	0.8505	SVC
Tumor_peri1 (Ph6)	Tumor (T2FS)	36	0.8825	0.9207	0.9130	0.8824	SVC
Tumor_peri1 (Ph6)	Peri1 (T2FS)	48	0.8641	0.9196	0.8406	0.8655	SVC
Tumor_peri1 (Ph6)	Peri3 (Ph2)	23	0.8986	0.8890	0.8551	0.8688	Logistic
Tumor_peri1 (Ph6)	Peri3 (T2FS)	41	0.8779	0.9130	0.9130	0.9130	Random Forest
Tumor_peri1 (Ph6)	Tumor_peri3 (T2FS)	38	0.8802	0.9130	0.9130	0.9130	Random Forest
Peri3 (Ph2)	Tumor (Ph6)	35	0.8836	0.9171	0.8261	0.8547	Decision Tree
Peri3 (Ph2)	Tumor (T2FS)	59	0.8318	0.8915	0.7826	0.8199	Random Forest

Peri3 (Ph2)	Peri1 (Ph6)	23	0.8986	0.9335	0.8986	0.9098	Random Forest
Peri3 (Ph2)	Peri1 (T2FS)	51	0.8594	0.8785	0.8116	0.8374	Random Forest
Peri3 (Ph2)	Peri3 (Ph6)	47	0.8652	0.8915	0.7826	0.8199	AdaBoost
Peri3 (Ph2)	Peri3 (T2FS)	16	0.9090	0.9281	0.7536	0.8016	AdaBoost
Peri3 (Ph2)	Tumor_peri3 (Ph6)	62	0.8249	0.8642	0.8261	0.8425	Random Forest
Peri3 (Ph2)	Tumor_peri3 (T2FS)	46	0.8687	0.8915	0.7826	0.8199	Random Forest
Tumor_peri3 (T2FS)	Tumor (Ph2)	28	0.8940	0.9329	0.9275	0.9088	SVC
Tumor_peri3 (T2FS)	Tumor (Ph6)	73	0.7857	0.8890	0.8551	0.8688	Random Forest
Tumor_peri3 (T2FS)	Peri1 (Ph6)	34	0.8848	0.9188	0.8986	0.9062	Random Forest
Tumor_peri3 (T2FS)	Peri3 (Ph6)	69	0.7995	0.8872	0.7536	0.7985	Random Forest
Tumor_peri3 (T2FS)	Tumor_peri3 (Ph6)	74	0.7834	0.8609	0.8116	0.8324	Random Forest
Tumor (Ph2)	Tumor (Ph6)	67	0.8018	0.8261	0.8261	0.8261	Random Forest
Tumor (Ph2)	Tumor (T2FS)	54	0.8571	0.8798	0.6957	0.7549	Logistic
Tumor (Ph2)	Peri1 (Ph6)	3	0.9401	0.9137	0.8841	0.8950	AdaBoost
Tumor (Ph2)	Peri1 (T2FS)	36	0.8825	0.8934	0.8696	0.8795	Logistic
Tumor (Ph2)	Peri3 (Ph6)	61	0.8272	0.8074	0.8986	0.8505	SVC
Tumor (Ph2)	Peri3 (T2FS)	25	0.8963	0.8981	0.9130	0.8970	SVC
Tumor (Ph2)	Tumor_peri3 (Ph6)	57	0.8410	0.8851	0.8406	0.8582	Random Forest

Peri1 (Ph6)	Tumor (T2FS)	51	0.8594	0.9207	0.9130	0.8824	SVC
Peri1 (Ph6)	Peri1 (T2FS)	59	0.8318	0.8915	0.7826	0.8199	SVC
Peri1 (Ph6)	Peri3 (T2FS)	25	0.8963	0.9021	0.8406	0.8624	Random Forest
Peri3 (Ph6)	Tumor (T2FS)	66	0.8111	0.8933	0.6087	0.6847	SVC
Peri3 (Ph6)	Peri1 (T2FS)	50	0.8618	0.9234	0.9275	0.9250	Random Forest
Peri3 (Ph6)	Peri3 (T2FS)	70	0.7972	0.8582	0.5217	0.6100	Logistic
Peri3 (T2FS)	Tumor (Ph6)	67	0.8018	0.8915	0.7826	0.8199	Random Forest
Peri3 (T2FS)	Tumor_peri3 (Ph6)	75	0.7097	0.8148	0.7681	0.7900	Random Forest
Tumor (T2FS)	Tumor (Ph6)	63	0.8226	0.8872	0.7536	0.7985	Random Forest
Tumor (T2FS)	Tumor_peri3 (Ph6)	72	0.7880	0.8725	0.8551	0.8629	LGBM
Peri1 (T2FS)	Tumor (Ph6)	64	0.8203	0.8922	0.8986	0.8951	Random Forest
Peri1 (T2FS)	Tumor_peri3 (Ph6)	65	0.8157	0.9207	0.9130	0.8824	SVC

Abbreviations: Ph2, second-post contrast subtraction image; Ph6, sixth-post contrast subtraction image; T2FS, T2 weighted fat-saturated image, Peri1, peritumoral region, 1mm; Peri3, peritumoral region, 3mm Tumor_peri1, tumoral + 1mm peritumoral region; Tumor_peri3, tumoral + 3mm peritumoral region; SVM, Support Vector Machine; LGBM, Light Gradient-Boosting Machine.

Table S4. pCR prediction performances of the clinical models in the validation cohort

Features	AUC	Precision	Recall	F1-score	Model
Age	0.6261	0.8766	0.7750	0.8180	K Neighbors Classifier
Tumor size	0.8063	0.9067	0.7500	0.8063	Logistic Regression

Age + Tumor size	0.6667	0.8936	0.6000	0.6933	Logistic Regression
ER expression	0.6847	0.8708	0.7250	0.7847	Decision Tree Classifier
PR expression	0.6396	0.8680	0.7000	0.7675	Random Forest Classifier
ER + PR expression	0.5315	0.8766	0.7750	0.8180	K Neighbors Classifier

Abbreviations: ER, estrogen receptor; PR progesterone receptor
